# Draft Genome Sequence of *Geobacter* sp. Strain SVR, Isolated from Antimony Mine Soil

**DOI:** 10.1128/MRA.00461-20

**Published:** 2020-06-25

**Authors:** Tomoro Warashina, Masafumi Harada, Nobuyoshi Nakajima, Shigeki Yamamura, Masaru Tomita, Haruo Suzuki, Seigo Amachi

**Affiliations:** a Institute for Advanced Biosciences, Keio University, Tsuruoka, Yamagata, Japan; b Systems Biology Program, Graduate School of Media and Governance, Keio University, Fujisawa, Japan; c Faculty of Environment and Information Studies, Keio University, Fujisawa, Kanagawa, Japan; d Center for Environmental Biology and Ecosystem Studies, National Institute for Environmental Studies, Tsukuba, Ibaraki, Japan; e Center for Regional Environmental Research, National Institute for Environmental Studies, Tsukuba, Ibaraki, Japan; f Graduate School of Horticulture, Chiba University, Matsudo, Chiba, Japan; Georgia Institute of Technology

## Abstract

We report here the draft genome sequence of *Geobacter* sp. strain SVR, isolated from antimony mine soil in Hyogo Prefecture, Japan. The genome sequence data in this study will provide useful information for understanding bacterial antimonate reduction.

## ANNOUNCEMENT

Antimony (Sb) and its compounds have been used for various industrial and commercial applications. However, Sb is considered a pollutant of high priority in developed countries (1). Knowledge about the molecular mechanisms of antimonate [Sb(V)] reduction by microorganisms is still very limited (2). Here, we report the whole-genome shotgun sequence of strain SVR, isolated from antimony mine soil in Hyogo Prefecture, Japan.

A strict anaerobic technique was used in the preparation of the minimal medium and the manipulation of the enrichments. The medium used in this study contained the following (per liter): NH_4_Cl (0.535 g), KH_2_PO_4_ (0.136 g), MgCl_2_·6H_2_O (0.204 g), CaCl_2_·2H_2_O (0.147 g), trace mineral element solution (1 ml), vitamin solution (1 ml), Se/W solution (1 ml), 1 g liter^−1^ resazurin solution (1 ml), and NaHCO_3_ (2.52 g). Acetate (5 mM) and Sb(V) (5 mM) were added as the sole electron donor and acceptor, respectively. An Sb(V)-reducing enrichment was prepared by inoculating a soil sample collected from a former Sb mine in Hyogo Prefecture, Japan. After several rounds of subculturing, the microbial community in the enrichment was analyzed by PCR-denaturing gradient gel electrophoresis (PCR-DGGE) targeting the 16S rRNA gene. Bacteria closely related to *Geobacter* spp., *Enterobacter* spp., and Citrobacter freundii were predominant in the enrichment. The enrichment culture was serially diluted and inoculated into anaerobic shake tubes solidified with Bacto agar. After repeated single-colony picking, an anaerobic bacterium closely related to *Geobacter* spp. was isolated and designated strain SVR.

Cells of strain SVR were grown in the minimal medium described above. Genomic DNA was extracted using a DNeasy blood and tissue kit (Qiagen, Hilden, Germany). The genomic DNA was sheared to 550 bp using the Covaris platform. Then, the library was constructed using the TruSeq DNA PCR-free LT library prep kit according to the supplier’s manual. Whole-genome sequencing was performed using the MiSeq sequencing platform (Illumina, San Diego, CA) at the National Institute for Environmental Studies. The sequencer produced 1,397,053 paired-end reads (2 × 300 bp).

The paired-end reads were processed as described elsewhere (3). Default parameters were used for all software unless otherwise noted. Briefly, phiX contamination was removed using BBDuk (https://sourceforge.net/projects/bbmap/). After that, the reads were filtered to remove adapter sequences and low-quality and short (<100 bp) reads using Trim Galore version 0.4.5 (https://www.bioinformatics.babraham.ac.uk/projects/trim_galore/). The quality of the reads was checked using FastQC version 0.11.5 (4). *De novo* genome assembly was performed using Unicycler version 0.4.7 (5). After possible contaminant sequences were removed, the resulting assembly contained 45 contigs totaling 4,662,216 bp, with a G+C content of 58.0% and 162× average genome coverage. The quality of the genome assembly was assessed using QUAST (6). The largest contig was 546,751 bp, and the *N*_50_ and *L*_50_ values were 233,456 bp and 7, respectively. Genome annotation was performed using DFAST-core version 1.1.2 (7), yielding 4,356 protein coding DNA sequences (CDSs), of which 1,712 (39%) are proteins of unknown function annotated as “hypothetical proteins.”

Strain SVR was the first Sb(V)-reducing bacterium in the genus *Geobacter.* The detailed characteristics of Sb(V) reduction by this strain will be reported elsewhere. The genome sequence data in this study will provide useful information for understanding bacterial antimony reduction.

### Data availability.

The genome sequence of *Geobacter* sp. strain SVR has been deposited as a whole-genome shotgun project at DDBJ/EMBL/GenBank under BioProject accession number PRJDB5044, BioSample number SAMD00154418, and accession number BJBU00000000 (BJBU01000001 to BJBU01000045). The version described in this paper is the first version, BJBU01000000. The raw data have been deposited in the DDBJ Sequence Read Archive (DRA) under accession number DRR165663.
